# A practical guide to cross-cultural and multi-sited data collection in the biological and behavioural sciences

**DOI:** 10.1098/rspb.2023.1422

**Published:** 2024-04-24

**Authors:** Laure Spake, Anushé Hassan, Susan B. Schaffnit, Nurul Alam, Abena S. Amoah, Jainaba Badjie, Carla Cerami, Amelia Crampin, Albert Dube, Miranda P. Kaye, Renee Kotch, Frankie Liew, Estelle McLean, Shekinah Munthali-Mkandawire, Lusako Mwalwanda, Anne-Cathrine Petersen, Andrew M. Prentice, Fatema tuz Zohora, Joseph Watts, Rebecca Sear, Mary K. Shenk, Richard Sosis, John H. Shaver

**Affiliations:** ^1^ Binghamton University (SUNY), Binghamton, NY, USA; ^2^ London School of Hygiene and Tropical Medicine, London, UK; ^3^ Pennsylvania State University, University Park, PA, USA; ^4^ International Centre for Diarrhoeal Disease Research, Bangladesh (icddr,b), Dhaka, Bangladesh; ^5^ Malawi Epidemiology and Intervention Research Unit, Lilongwe, Malawi; ^6^ Leiden University Medical Center, Leiden, The Netherlands; ^7^ Medical Research Council Unit The Gambia at the London School of Hygiene and Tropical Medicine (MRCG@LSHTM), Fajara, The Gambia; ^8^ University of Glasgow, Glasgow, UK; ^9^ University of Chicago, Chicago, IL, USA; ^10^ University of Otago, Dunedin, New Zealand; ^11^ University of Canterbury, Christchurch, New Zealand; ^12^ University of Connecticut, Storrs, CT, USA; ^13^ Baylor University, Waco, TX, USA

**Keywords:** research design, multi-site research, open science, study planning, study protocol, cross-cultural research

## Abstract

Researchers in the biological and behavioural sciences are increasingly conducting collaborative, multi-sited projects to address how phenomena vary across ecologies. These types of projects, however, pose additional workflow challenges beyond those typically encountered in single-sited projects. Through specific attention to cross-cultural research projects, we highlight four key aspects of multi-sited projects that must be considered during the design phase to ensure success: (1) project and team management; (2) protocol and instrument development; (3) data management and documentation; and (4) equitable and collaborative practices. Our recommendations are supported by examples from our experiences collaborating on the Evolutionary Demography of Religion project, a mixed-methods project collecting data across five countries in collaboration with research partners in each host country. To existing discourse, we contribute new recommendations around team and project management, introduce practical recommendations for exploring the validity of instruments through qualitative techniques during piloting, highlight the importance of good documentation at all steps of the project, and demonstrate how data management workflows can be strengthened through open science practices. While this project was rooted in cross-cultural human behavioural ecology and evolutionary anthropology, lessons learned from this project are applicable to multi-sited research across the biological and behavioural sciences.

## 1. Introduction

Researchers are increasingly employing multi-sited research to build more robust understandings of social, behavioural and biological phenomena across ecologies [1–4]. Cross-cultural research is a specific case of multi-sited research that explicitly considers cultural variation in its study design. Conducting multi-sited and cross-cultural research is a complicated endeavour. These types of projects entail challenges not typically encountered in single-team/single-site research, for example large international research teams, members of which are often accustomed to working independently, team members from different disciplines with a range of research expertise and preferred workflows, and instruments that must be effective and appropriate across several languages and cultural settings.

Recommendations for cross-cultural research in the evolutionary behavioural sciences are beginning to appear in the literature. Some authors raise critical issues associated with cross-cultural work such as selection of populations to study [2,5,6], validity of measurement tools and experimental protocols [5–7], project and team management [2,8], or equitable sharing of power in research partnerships [9]. Other teams have written more holistically about the process of conducting cross-cultural research, for example by identifying key challenges facing cross-cultural research teams at all stages of their projects and proposing solutions to overcome these [2,10–14]. Authors have covered topics spanning research design, data collection workflows, analytical processes, results dissemination, and collaborative practices, often weaving in discussions of ethical issues as they relate to each of these topics. Outside of the behavioural sciences, discussion on the process of collaborative and team-based science, especially from a team management perspective, is growing in biology and ecology as teams increasingly engage in multi-sited research projects [1,3,15]. Nevertheless, very few papers clearly outline the full process of cross-cultural data collection to guide researchers designing their own studies.

In this paper, we take a holistic approach, describing the workflows employed by our team to manage our ongoing cross-cultural project: the Evolutionary Dynamics of Religion, Family Size, and Child Success (henceforth, the EvoDemReligion project). This multidisciplinary, mixed-method study collected survey data from nearly 6000 participants and focus group data from about 500 participants living in five countries, with the aim of elucidating the relationships between religion, social support and cooperation, and maternal and child wellbeing. Our purpose in writing this paper is to: (1) highlight key challenges inherent to managing and designing cross-cultural research projects; (2) describe the workflows and solutions that can be deployed to manage these challenges; and (3) illustrate these solutions with examples from our own experiences. It is our hope that providing the technical details of our workflows will help others embarking on cross-cultural work to structure their own processes.

We begin by describing the project and its structure, highlighting that our project shared many of the features of typical cross-cultural projects in anthropology and the evolutionary behavioural sciences, although it is somewhat unusual in these disciplines in that it was carried out in close collaboration with teams from Health and Demographic Surveillance Systems (HDSS). We then focus on four key aspects of the cross-cultural research process that pose organizational challenges and explain how our team addressed them. Specifically, we discuss: (1) team and project management; (2) protocol development; (3) data management and documentation; and (4) equitable and collaborative practices. Throughout this discussion, we highlight the roles that collaborative practices and open science principles played in ensuring the success of the project. In closing, we consider how our workflows respond to recent calls by researchers for high-quality cross-cultural research practices [12], reflect on the strengths and weaknesses of our processes, and summarize our recommendations for other teams developing their own cross-cultural research framework. Though we speak primarily from the perspective of behavioural science research, lessons learned from our work extrapolate well onto multi-sited research in cognate disciplines including biology, psychology, and ecology.

### (a) The Evolutionary Dynamics of Religion, Family Size and Child Success project

The overall aim of our project was to investigate how religiosity affects fertility, social support and cooperation between community members, and how these, in turn, affect wellbeing outcomes for women and children across socioecologies (see our project OSF page: https://osf.io/mztep/). Data collection for the project took place in five countries: Bangladesh, The Gambia, India, Malawi and the USA. These countries were selected in a principled way to ensure that settings varied in several indicators of interest to our study, specifically: religion, religiosity, and religious practices, fertility rates and schedules, geographical region and levels of market integration [5,10,11,16]. Selecting study locations that vary along these continua enables us to compare the role of our independent variables (religiosity indicators) under varying sociodemographic conditions (e.g. number of dependent children and residential proximity to kin may impact how religiosity acts to help parents access social support). Practical considerations were also factored into the decision: we largely (though not exclusively) opted to work in some locations where our team had prior research relationships or could leverage institutional relationships to build partnerships with local research organizations. Still, three of the five collaborations with partner institutions that conducted this project were new. The EvoDemReligion project was supported by funding from the John Templeton Foundation and the Templeton Religion Trust. Importantly, the project was initially supported through a planning grant which supported literature review, dialogue with topic experts, development of hypotheses, relationship-building with research partners, and secondary analyses in order to plan cross-cultural data collection.

The project team was large and spread across seven countries and many institutions. The core team responsible for the development and coordination of the project consisted of four principal investigators, a team of post-doctoral researchers, and project coordinators. Several other researchers were also involved in the core team but focused on other aspects of the project such as theoretical modelling and secondary data analyses. Within each study country, a team of local collaborators contributed to various aspects of the study through scientific contribution, data management and instrument coding, project management, data collection, and/or leadership of interviewer teams.

**Figure 1.**
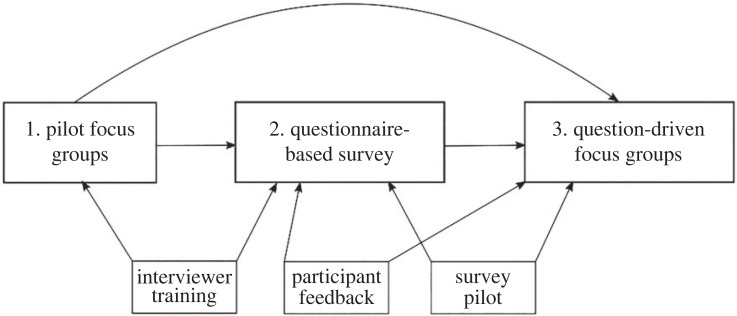
Diagram of the research components (1–3). Arrows indicate the application of information learned from one component to revise other components.

The mixed methods study design consisted of two sets of focus group discussions and long-form surveys run through Open Data Kit (ODK), an open-source survey software (https://getodk.org/; figure 1). The initial set of focus groups was aimed at understanding local variations in key indicators. For example, we asked questions about types of childcare and other social support typically performed and exchanged in the community, about religious practices and how they varied across gender, age, or religiosity and marriage and family planning ideals. The information gained from these discussions was used to adapt standardized questionnaires to be locally appropriate. Questionnaires were then piloted and adjusted until the interviewers were satisfied with their performance. In other words, we piloted until interviews flowed smoothly, all errors in questions and/or answer choices had been fixed, and participants no longer reported confusion with the survey questions. The process of piloting took roughly one month and included modifications made after focus group discussions, during interviewer training, and while piloting the survey. Due to COVID-19 restrictions, piloting did not occur simultaneously in each country as originally intended. Thus, the piloting stage in the first countries to start data collection took longer than in the countries which began later, as the latter benefited from more major revisions made in the former. After the initial focus groups and questionnaire piloting, we revised discussion guides for the second set of focus group discussions to gain deeper understanding of the survey topics.

Features of our team and project impacted the workflows we adopted and are worth presenting here. First, as in many large research teams, there were multiple team members in similar roles with similar technical expertise, although spread across several institutions. This resulted in the need to define and coordinate roles, particularly amongst the post-doctoral team who was responsible for daily project management. However, while there was overlap in technical expertise amongst our team, one of our strengths was the diversity in topical expertise spanning human biology, biological anthropology, demography, evolutionary anthropology, and human behavioural ecology. Second, our collaborators within each study country were primarily based at institutions hosting HDSS (with the exception of the US team). Although HDSS are well versed in international scientific collaboration, these collaborations are somewhat unusual in anthropology. In our case, HDSS collaborations facilitated our work, as we benefited from their research expertise and strong, on-going relationships with communities within the study areas (see Aspect 4). Since HDSS maintains records of all inhabitants of a region, working with them also facilitated participant sampling and recruitment. Our experiences with the EvoDemReligion project, and thus the recommendations we make in the following section, are coloured by these team and project features.

## 2. Workflows for organizing aspects of cross-cultural research projects

Cross-cultural research, and multi-sited projects more generally, present several unique challenges due to the nature of the work. Accordingly, in the planning and development phases of such projects, teams should consider several aspects crucial to success. In this section, we address each of these aspects in turn. For each aspect, we offer context, make recommendations for structuring related workflows, and support these recommendations with our experiences.

### (a) Aspect 1: project and team management

Cross-cultural research projects are inherently collaborative, often with teams spanning countries and institution types—universities, government, NGOs, and others. While such collaboration is a major strength of these projects, it also comes with coordination costs to productivity [15,17]. Coordination costs can include time-consuming challenges such as conflicting goals, communication difficulties, bridging gaps between different disciplinary norms, or establishing new working processes and routines [17,18]. Team members in collaborative projects are often asked to juggle additional managerial responsibilities (e.g. international communication) with existing responsibilities including research, institutional work or teaching, which has implications for job satisfaction, productivity, and wellbeing [17,18]. This is especially true for collaborators who are managing projects on the ground, and are often asked to coordinate between projects with little or no additional financial, training or staffing support (see Aspect 4). Coordination costs tend to be largest at the onset of projects while teams are still learning how to work together, but can also increase whenever there is turnover in a team. Managing these costs appropriately minimizes loss of productivity through the life of the project.

Coordination costs for the EvoDemReligion project were exacerbated by the COVID-19 pandemic. The pandemic delayed and desynchronized all timelines—for hiring and research—as different countries experienced the pandemic and its repercussions differently. Some of the key coordination costs faced by our team included: negotiating roles and responsibilities of team members entering the project at different times, establishing centralized communication systems between team members with access to different institutional tools, and creating meaningful engagement with team members who were less involved on a day-to-day basis. Though these coordination challenges are nearly universal in cross-cultural research (even without a pandemic), few have reported on the importance of efficient and clear project management (for an exception, see [2]). Below, we detail three easily adoptable coordination activities our team implemented to increase coordination amongst our members: (1) establishing clear roles and responsibilities; (2) establishing open and centralized channels of communication; and (3) additional knowledge sharing and decision-making meetings.

#### (i) Establishing roles and responsibilities

Establishing roles and responsibilities amongst collaborators is an essential precursor for all further project management [18]. The post-doctoral team was responsible for daily management of the project and for working with research partner institutions and local collaborators across different study locations. Since their roles were very similar the post-doctoral team explicitly negotiated individual roles and met regularly to allocate tasks and responsibilities. This limited duplication of work and prevented tasks from ‘falling through the cracks' due lack of clear leadership. Creating a working structure with clear decision-making procedures and actionable goals ensured that each team member knew what their tasks were and the team was able to re-assign tasks when necessary.

#### (ii) Establishing communication channels

Early in project development, the post-doctoral team established communications procedures. We used Microsoft Teams, which enables a working group to collaborate on version-controlled documents, to message and video call, and allows linking to secure cloud storage through Microsoft OneDrive and Microsoft SharePoint. Version-controlling documents in a centralized storage platform ensured that modifications were never made to an outdated version of a document, and we never had to dig through e-mail communications to find a specific draft. Additionally, sharing and centralizing all project-related documents meant we could easily onboard new researchers when needed by sharing access to the platform (see §2d(iv)).

However, using proprietary software to host files can complicate cross-institutional collaboration. For example, over the course of the project team members frequently experienced difficulty signing into Microsoft Teams due to cross-institutional login challenges, and we found it did not work well during some study location visits because of poorer Internet bandwidth. Alternative software, both institutionally-subscribed and free, e.g. Google Docs, Dropbox, and Overleaf, may be better options although the last option is only suitable for text processing files. An alternative for centralizing team communications might be Slack, which in its free version allows one-to-one video calls and integrates with both Google Drive and the Microsoft suite (SharePoint and/or OneDrive) for document collaboration. However, free versions of such software tend to have limitations; for example, Slack's free version only archives messages for 90 days, after which they are permanently deleted. Additionally, while these tools are used in many parts of the world, they are not commonplace in others. Training and support may be necessary to help some collaborators become comfortable using these tools, and training may need to be renewed as tools change.

In deciding which communications solutions are appropriate, we encourage researchers to consult their institutions about data security and privacy protocols. Technology is becoming increasingly important for cross-institutional collaboration, but institutions do not all subscribe to the same services. As freeware can have significant limitations, research teams may want to budget for software purchases and/or subscriptions in their funding applications. Teams may also want to plan for costs associated with in-field connectivity such as 4G Internet routers, SIM cards and subscriptions, and solar panels or generators if necessary. Lastly, productivity tools only work if team members are willing to use them; our advice would be to prioritize tools that are familiar to team members and ideally already in use.

#### (iii) Additional coordination activities

We held several knowledge-sharing coordination activities throughout the life of the project. Knowledge-sharing activities are situations where learning and knowledge production occur between team members (e.g. seminars, co-writing papers), and these activities are shown to reduce the costs to productivity of working in large project teams [18]. Several times per year, our core team held meetings to share updates on the status of work at each study location and to make key decisions on the future of the project. We also organized a speaker series comprising public talks (recorded and subsequently uploaded online) during which we shared results of in-progress research. These talks stimulated discussion amongst our team and with external researchers, resulting in new analytical directions. We would encourage teams to meet regularly throughout the project lifecourse in appropriate ways to coordinate and share early results.

### (b) Aspect 2: protocol and instrument development

Perhaps the most often and highly discussed aspect of cross-cultural research is protocol and instrument development. In multi-sited and cross-cultural research projects, the key challenge is striking the right balance between instrument standardization across sites and accounting for known variation between sites in culture, research infrastructure, laws, etc. [5,6,11,19]. Data collection tools specifically are difficult to standardize. Tools regularly used in one context may be unfamiliar to participants in other contexts [7], which can slow down interviews and potentially create noise in the data. For example, Likert-type items, in which participants rate their agreement with a statement on a 5- or 7-point scale, are difficult to use and compare cross-culturally [2,7,20,21]. We experienced this in the EvoDemReligion project: some Likert-scale questions were not understood by participants in some contexts, while in others they showed little variation in response and were ultimately removed from the survey.

In this section, we focus on processes that can help teams ensure their protocols and instruments are valid across culturally diverse study settings. Cross-cultural research spans many types of research methods including in-depth interviews, questionnaires, economic games, and other experimental designs. Consulting the literature provides advice on developing some types of cross-cultural research tools [11,12,19]. More universally, processes that can help researchers ensure their protocols and data collection tools are cross-culturally valid are (1) pre-registration; (2) obtaining early input from in-country collaborators; (3) a well-planned piloting phase; and (4) implementing standard operating procedures.

#### (i) Pre-registration of main research questions, predictions, hypotheses

A pre-registration document outlines a study's hypotheses, methods, and analytical approach prior to the onset of data collection [22,23]. It is typically used to increase transparency and reduce the potential that a research team will knowingly or unknowingly engage in questionable research practices [22]. The act of creating a pre-registration document is also an excellent opportunity for a team to discuss and agree upon project aims, methods, and analytical approaches [23]. Creating a pre-registration document encouraged our team to not only agree upon our key predictions but also start discussions about operationalization of key concepts and how the data would ultimately be analysed. Additionally, the pre-registration became a document to continually refer to when designing, piloting, and modifying our instruments to ensure that our data collection tools measured each prediction's key variables.

We approached pre-registration from a modular perspective: different components of the project were pre-registered separately [24]. Before data collection began, our team drafted our first pre-registration which included: our main research questions, each derived from theory and accompanied by a set of predictions; a dictionary of key indicators; a basic study design; and a short analysis plan for both the quantitative and qualitative components of the project. More specific analyses were pre-registered after data collection was complete and are nested within the original, broader registration. This modular approach to pre-registration was originally proposed for longitudinal studies [24], but lends itself well to multi-sited research where changes to instrument and methods are made during piloting. We encourage teams to complete pre-registration documents prior to data collection and recommend a modular approach.

#### (ii) Construction of standardized instruments with early input from local collaborators

The expertise of scientific collaborators at each partner institution, who are scientists from the study countries, have worked with the communities for many years, and/or speak the local language(s), was vital during our instrument design phase (see electronic supplementary material, figure S1, for our design workflow). Their expertise shaped the project and its data collection procedures early on. For example, residents of West Kiang HDSS in The Gambia live in compounds that can count over 100 residents. Our collaborators recommended we revise household rostering procedures to reduce our questionnaire length. In some cases, scientific collaborators pointed us towards resources or relevant work from other scholars to help us better understand local contexts. This resulted in improved adaptations of questionnaires to each context and reduced the number of revisions needed during piloting. For additional ethnographic insights while designing questionnaires, researchers can consult the Human Relations Area Files (eHRAF, https://ehrafworldcultures.yale.edu/), country-specific Demographic and Health Surveys (https://dhsprogram.com/), or published research (ethnography or otherwise). We would strongly suggest that teams seek out local collaborators early in the research design phase and that they be invited and supported to provide meaningful contribution to the study design (see Aspect 4).

#### (iii) Planning a pilot to explore local instrument validity

The pilot phase is used to field-test, revise, and then fine-tune data collection processes and instruments. Pilot phases are generally intensive, entailing multiple rounds of rapid feedback and revision to instruments. This process can be exhausting, but pressure can be reduced if the pilot is well planned. Based on our experiences, we suggest that: (1) pilots be timed well ahead of data collection; (2) researchers budget significant time, generally 3–4 weeks for detailed surveys, especially if working in a new study location; (3) researchers have an explicit plan for adapting and updating instruments to reflect local cultural norms; and (4) pilots be conducted in all study locations.

Firstly, we recommend separating the piloting and data collection phases temporally. Ideally, we suggest researchers make two separate trips (one for piloting and one for data collection), a suggestion others have also made [2]. Separating the two phases allows the full project team to meet to review changes to the data collection protocols, which can be difficult to do with poor Internet connections and/or time pressures in the field. Separating the two phases also allows for time to submit amendments to project protocols for reviews by ethics boards where necessary. However, making two separate trips is costly financially, in terms of time, and in environmental impact in a warming world. Where two separate trips are not preferable, an alternative approach would be to implement a buffer period (e.g. 1–3 weeks) between the pilot and data collection phases, or the time needed to submit and receive approval for an ethics amendment. This buffer period should include scheduled meetings with all project team members for consultation on protocol modifications, with a view on how changes may impact protocol validity at other study locations. Building in this pause between phases is particularly important for research teams piloting in areas with limited Internet and/or phone connectivity, and allows researchers a chance to rest and carefully check instruments for any remaining errors.

Secondly, we suggest that researchers budget more time for piloting than they anticipate needing, particularly if working in a new study location. This is because working with partner institutions and communities means developing relationships that take time to form. For example, in one study location, local procedures required conducting meetings to explain the project and request permission to conduct the study in each participating village. These meetings improved our data collection protocols and relationships with local communities but required additional time at the onset of data collection. Likewise, interviewer training tended to take longer than anticipated, though it was highly valuable as interviewers flagged errors in the questionnaire and offered suggestions for adapting questions to be more locally relevant. Longer pilot phases must be recognized by funding agencies and research institutions alike as an integral component of multi-sited projects. Funders should commit to funding multi-site projects for longer periods of time than single-site projects.

Thirdly, we recommend that researchers make an explicit plan for adapting and updating instruments to reflect local cultural norms, ideally using qualitative methods [12]. To do this, our team adopted a two-pronged qualitative approach. First, we conducted a series of initial focus groups aimed at exploring local conceptions of key variables of interest. Immediately following each focus group, interviewers summarized the content of the discussion and in the following days orally translated the discussion recordings. This information helped us adapt sections of our surveys we had identified as needing locally relevant examples and questions. For example, our surveys asked questions about the receipt of key types of childcare support. In the USA, focus group discussions revealed carpooling as a key form of childcare support, but carpooling was irrelevant in other study countries. Second, we used a cognitive interview method during the pilot phase, in which interviewers probe participants' thoughts and feelings as they complete a questionnaire [7,25]. Specifically, interviewers were asked to record any questions or requests for clarification, and any reactions to specific questions, posed by participants during piloting, and to ask the participants why they reacted to the question in this way. For example, mothers in The Gambia found the question about playing with school-aged children funny. They told interviewers that these children were too old to play with, and instead mothers spent time with them by sitting together, chatting, and teasing each other. We modified questionnaires to reflect this information. All piloting forms, including the interviewer impression recording forms, are available on the project OSF page (https://osf.io/mztep/).

Finally, we recommend conducting pilots in multiple, if not all, study locations prior to finalizing the study protocol and instruments. Ideally, pilot locations should have differing socioecologies to ensure that the protocols will work in different contexts. Study locations less familiar to the project team are also well suited for piloting, as the team is less able to anticipate what might work in these places. In our case, our initial pilot was conducted simultaneously in two of the five locations due to COVID-19 related delays, but we were able to consult at this stage with collaborators who had worked long-term in another study location (Bangladesh), and/or were familiar with the study area (USA). We could therefore anticipate how modifications to our instruments might impact data collection in the locations where piloting took place later on.

#### (iv) Standard operating protocols

Standard operating procedure (SOP) documents can help ensure consistency in data collection procedures. Our project benefited from creating SOPs, which helped structure interviewer training and served as a reference throughout the project. Our 71-page final document covered a range of topics including study background, an outline of each component of the project, participant recruitment procedures, daily data collection procedures, and a set of responsibilities for the interviewer team supervisor. The SOPs also contained, as a series of appendices, all documents associated with data collection: focus group guides, participant information sheets, forms to be completed by interviewers when visiting households, a guide for the questionnaire software (ODK), and procedures for collecting anthropometrics. Our SOPs are available on the project OSF page. Writing SOPs is time consuming but ultimately beneficial: it nudged us to think through the details of daily workflows, echoing how the pre-registration document prompted detailed conversations about methodology. Working through these documents helped prepare us for interviewer training, and ensured we used standardized procedures across each of the five study locations. Producing such documents also helps ensure that researchers working across locations are consistent in participant selection and data collection procedures, which can be a challenge [2].

### (c) Aspect 3: data management and documentation

Multi-sited and cross-cultural projects yield large amounts of complex data including surveys, videos, audio recordings, biospecimens, data from wearable devices, and/or notes in electronic or hand-written formats. In our case, at the peak of data collection, we were receiving over 100 survey submissions daily. Managing and checking this amount of data require clear processes and procedures [12]. In keeping with open science principles, workflows for monitoring, managing, and manipulating data should be reproducible and ideally automated to increase efficiency [26,27]. Data workflows require technical skills, which may require training team members or hiring specialists, something that investigators need to budget for in grant proposals. Data workflows should ideally be agreed upon ahead of time, as these can affect the quality and reliability of data [26]. Researchers should agree upon workflows for (1) quality assurance and quality checks (QAQCs); (2) data processing, transformation, and analysis; and (3) long-term data storage and security protocols.

#### (i) Quality assurance and quality checks

Examples of QAQC workflows include processes that check for data entry errors, nonsensical answers, and repeated errors during interviewing or data entry. Our team used tablet-based questionnaires, which reduced data-entry time and transcription errors and allowed the core team to have access to data as they were collected. However, data entry errors in dates of birth or identifying information did occur during the pilot—a significant concern with tablet surveys as there is no paper backup to refer to if data are entered incorrectly. To manage and ideally avoid these errors, we developed R scripts to monitor key questions during piloting and data collection and communicated recurring mistakes to interviewers. When possible, we also asked interviewers to return to households to correct errors. The scripts used to monitor data were shared amongst the research team using GitHub so they could be easily adapted to each study location. For programming-savvy teams, a QAQC script can be automated to run prior to integrating new data into the database, so that entries with errors are rejected and must be corrected prior to entry into the database [27]. Data monitoring through QAQC mechanisms, whether these are automated and developed ahead of time or performed manually at the start of data collection, can be quite time consuming. Teams should be prepared for the fact that personnel assigned to QAQC may find their time fully consumed by this task during and immediately following the data collection period.

#### (ii) Data processing, transformation, and analysis

**Figure 2.**
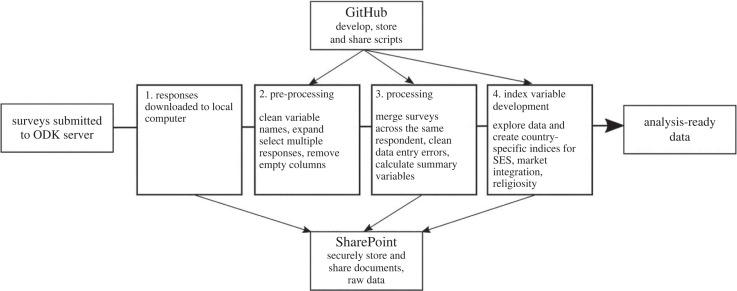
Illustration of the data processing workflow (steps 1–4), which was applied to each of the country-specific datasets.

All data processing should occur in programmatic environments, typically R, Stata, or Python, so that raw data are never edited directly. Point changes and variable transformations done by hand are problematic because they leave no record of changes and are prone to introducing irreversible errors [2]. Scripts serve as documentation of changes, allow transformations to be reversed or modified, and are time-saving in the long run. In our case, since data structures were similar in all study locations, we built data cleaning and processing scripts using data from one study location, then shared scripts among the team through GitHub so that they could be modified and applied to data from the other study locations (figure 2). When data errors were identified in our datasets, we collectively developed rules for fixing them based on our knowledge of the study community, data collection procedures, and the institutional procedures for collecting the dates of birth in our sampling frame, and applied them uniformly across datasets. Besides saving time by minimizing duplication of work, applying a template script to each dataset ensures that processing decisions are applied similarly across datasets.

#### (iii) Long-term data storage and security

Another issue that cross-cultural researchers should anticipate early on is long-term reuse of the data, either by project members or by external researchers [2]. Decisions about data storage and future use are typically part of ethics review processes, and teams should think carefully through their data management plans at this stage. This can and should include agreements or protocols for data sharing that specify what types of data (raw, anonymized, or none at all) can be shared, under what circumstances they should be shared, and who should approve requests for data sharing. Teams should also consider which data storage platforms they will use when developing funding proposals, as some may incur costs. Long-term storage solutions should be in place before data collection to avoid transferring data between storage locations later.

Raw and potentially identifiable data should be stored in secure locations approved by ethics review boards of all participating institutions. Data need to be stored such that they are accessible to all relevant team researchers, and protocols should be developed for whether and how researchers share data (and with whom). GitHub repositories, even when made private, are not considered secure storage and thus are inappropriate for raw data files. Our protocol involved storing raw and identifiable data on a private shared Microsoft SharePoint accessible only to project members. SharePoint was selected as it met the security requirements of university and partner institution ethics review boards.

Our team planned for long-term archiving of both quantitative and qualitative data. Some research teams, particularly those who code-share amongst team members, use GitHub for this purpose. However, current consensus across open science advocates is that this is not an adequate solution as GitHub repositories can easily be deleted at any time and Digital Object Identifiers (DOIs) cannot be assigned to reference the project [27,28]. Once our de-identified dataset is ready to share, we will reposit it on our OSF page to centralize all materials associated with our project. Other free repositories that can be considered include: Zenodo for data and documents, Figshare for documents, images, and large datasets, and protocols.io for protocols; however, note that some repositories may charge fees.

#### (iv) Documentation

Large-scale, multi-sited research project teams recognize the importance of documentation for several reasons [2,12]. In such projects, researchers typically analyse data they themselves did not collect. Additionally, long-term projects can expect turnover in team members throughout the project's lifespan [2]. Lastly, the large amount of data produced are often made available to external researchers for secondary analysis. These data must be well documented for both internal and external researchers to use. Due to this, we agree with Purzycki and colleagues [2] that project documentation should itself be treated as a deliverable—adequate time should be allocated to producing it.

Collecting, storing, and sharing of data, metadata, and protocols was an important outcome for the EvoDemReligion project. Once complete, all documentation files were uploaded to the project OSF page. This included pre-registration documents, research instruments (including questionnaire codebooks, focus group discussion guides and our SOP document), as well as data processing scripts. We also produced a data documentation file, which explains the less obvious parts of the project, e.g. sampling and recruitment procedures including rationale for any country-specific modifications, the structure of data files, and details of the data processing steps applied to them. These files made on-boarding of new researchers onto the team quick and easy, as they could use these documents to understand data structures and collection procedures.

A benefit of using structured communication practices (see Aspect 1) and open-science practices was that these facilitated the compilation of project documentation. For example, using open-access software to deliver our survey (the Open Data Kit suite, secure server provided by LSHTM Global Health Analytics) meant that we could use freeware to quickly produce codebooks (https://figured.io/odkCodebookApp/). Since our communication protocols included centralized file storage, it was easy to pull the latest versions of documents from our shared storage location, convert them to PDFs, and archive them onto our OSF project page. Similarly, using R scripts to manipulate our data from their raw form into usable formats allowed us to easily document the data processing steps applied to our dataset.

### (d) Aspect 4: equitable and collaborative practices

Cross-cultural research projects inherently involve relationships (a) amongst researchers and (b) between researchers and participants that, if not meaningfully created and maintained, can reflect and perpetuate power imbalances. These projects often involve researchers from high-income countries working with collaborators in low-income countries. Dynamics between researchers from these different settings are inherently power imbalanced, and are affected by a history of systems of colonialism [29,30]. One manifestation of these imbalances is the tendency for researchers from high-income countries to participate in ‘parachute research’, i.e. well-funded researchers study phenomena in less well-resourced settings without collaborating with local researchers, investing in local research capacity, or sharing the benefits of academic research with either local researchers or the communities where they work [9,29,30]. Even when well-funded researchers collaborate meaningfully with local researchers, these partnerships can be burdensome for local researchers if not carefully structured. For example, local collaborators are often asked to manage fieldwork on top of other commitments. This can add extra stress if it is not accompanied with meaningful financial and/or staffing support. Collaboration may even carry potential risk to local researchers and their institutions if the topic of research is culturally sensitive, or if it is not carried out with sufficient attention to local political realities [31]. It is imperative that cross-cultural researchers avoid working in extractive ways and instead shift to more collaborative models.

In addition to relationships amongst researchers, those between researchers and participants can also become extractive—that is, information is taken from study participants who receive nothing in return. Such a dynamic between researcher and participant again likely reflects and perpetuates inequitable structures arising due to many factors including wealth inequalities, differential access to education, classism, and legacies of colonialism. Participants may ask what tangible benefits will be gained through participation. Researchers can design their research process to engage meaningfully with the individuals who comprise the communities where research takes place. Meaningful engagement with stakeholders that results in co-production of scientific knowledge (e.g. [32,33]) is the ideal form of community-engaged research; however, we should also recognize that some communities may find co-production burdensome and prefer to simply participate instead. Not every project must be community-based participatory research, but all projects should strive to make themselves more community-engaged. One obstacle to community engagement is funding structures: funding agencies should provide more support for exploratory and pilot research that allows meaningful engagement with local communities to build the relationships necessary to co-produce knowledge and develop larger research proposals [34]. Funders could also promote engagement and collaboration by requiring proposers to include in their proposals a statement explaining how they intend to adopt equitable and collaborative practices in their work, similar in nature to data management plans currently required by many funding agencies. We suggest that regardless of their level of collaboration with communities, all teams should consider how they will: (1) incorporate and recognize the contributions of local collaborators and partner institutions and (2) strive towards greater community engagement. These aspects of the project should be considered prior to applying for funding and should be adequately budgeted for in funding proposals.

#### (i) Incorporating, recognizing, and supporting the contributions of local partners

Creating more equitable partnerships between researchers can be done in various ways including: developing long-term relationships between well-resourced and less well-resourced research institutions in which all researchers contribute to study design and are credited for their work through authorship; investing in research capacity in less well-resourced study sites through mentorship, training, and skills development for local collaborators as well as interviewers; and choosing to channel research through local research institutions so that they can benefit from the work [9,11,12,14]. At each study location, the EvoDemReligion core team collaborated with one or several scientific collaborators, who gave feedback on research design and initial versions of study instruments (see Aspect 2). The core team also typically collaborated with the data management team and/or the HDSS team to select a sampling frame for the study, create data management plans suitable to both the core team and the partner institution, and/or design data collection instruments. For many study locations, we directly collaborated with one or two project managers who helped us hire assistants, facilitated data collection, and obtained materials and resources necessary for the project. Each study location recruited a team of between eight and forty interviewers, led by one or more supervisors or coordinators who were responsible for managing daily work when researchers from the core team were not present. Interviewers were often but not exclusively members of the study communities, some of whom had been working with our partner institutions for many years. The interviewer teams provided meaningful feedback on study instruments across a range of topics, indicating where questions were unclear or where more appropriate questions could be substituted, where language could be updated, where key topics were missing, and/or where changes could be made to increase data collection efficiency.

In collaborating with partner institutions, the EvoDemReligion project used existing HDSS frameworks to recognize contributions of various individuals and/or departments to the project, including project managers, software and/or data teams, research officers, station directors, and/or human resources. This variously included paying salaries, authorship on presentations and publications, delivering training in data collection and/or analytical methods, investing in other practical training such as motorbike riding, and purchasing material resources that could be reused by interviewers and/or other projects (e.g. anthropometry equipment, computers, motorcycle protective equipment). To provide support to local collaborators, we asked core team members to be present for large portions of the pilot and data collection periods to assist with interviewer training, team management, procurement of supplies, and any other necessary tasks. For teams not working within the framework of an HDSS or similar organization, it is important to think about how contributions will be recognized ahead of time. Recognition of local contributions should be flexible to ensure that individual contributors are recognized with compensation that makes sense to them. The recent trend in some high-income country institutions to require co-authorship from collaborators in low-income study locations as a display of equitable partnerships may not always be helpful for, or even what is wanted by, the collaborators. For example, authorship may be important to contributors pursuing academic careers, while investing in resources such as job training, including research skills, and/or laptops may be more valuable to other contributors [9,11]. One way to stimulate these conversations could be through drafting an authorship agreement ahead of data collection to protect local collaborators. These agreements could specify which types of contributions constitute authorship, how project members become listed on publications (opt-in, opt-out, or other), and how authorship order will be determined, for example. Local collaborators should be involved early in discussions on recognizing contributions appropriately so that projects, and project budgets, can be planned accordingly.

#### (ii) Increasing community engagement

International research collaborations do not always consider how participants experience research, even though participants make research possible [29]. There are two simple and easily implemented strategies for increasing community engagement in projects: holding informational meetings before the start of a project and holding dissemination meetings at its conclusion. Beyond this, teams can choose to take more participatory approaches, where communities have a role in setting the agenda for the research or in interpreting results. Teams may also consider whether they can provide more tangible benefits to participants, for example by compensating participants for travel costs and/or time away from work incurred by participating in the study. Many HDSS provide tangible benefits to study area residents by providing access to health facilities, building referral systems for health issues found during research into project protocols, or mandating that research teams provide transportation to health facilities for these participants.

In the case of the EvoDemReligion project, we followed the lead of the partner institutions in designing our protocols. In some settings, we held informational meetings about the project in the villages in which we recruited participants prior to starting data collection. Such meetings are shown to be effective for disseminating information about the project even to individuals who do not attend the meetings themselves [35]. We would suggest that projects consider holding these meetings even if they are not customary in the study region [11]. These types of meetings are a form of relationship building that could help identify community interest in additional research and eventually lay the groundwork for co-produced research. In our meetings in The Gambia, for example, such meetings told us that several communities would like to see more research that involves men's concerns. Communities also requested that we inform them of the results of our project. Despite the fact that our project is ongoing, we have been able to meet this request by hosting community meetings to return initial findings in several study locations, and we are actively planning to return key findings to other communities we worked with. This can mean, and for us has meant, prioritizing this type of reporting over scientific publication after data collection. Return of results to participants in person is ideal, but in alternatives may be possible. For example, we are planning on producing flyers to disseminate to US participants, who were interviewed via Zoom. Committing to community engagement in research means planning of additional time and funds in research and funding proposals.

## 3. Discussion and conclusion

**Figure 3.**
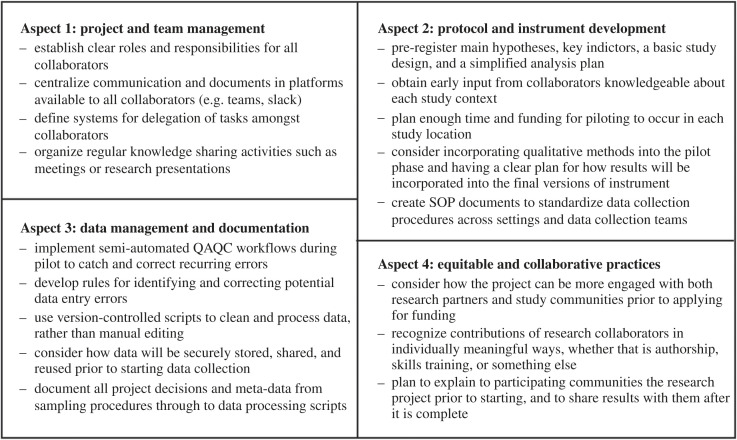
Summary of this article's recommendations on each of four key aspects of cross-cultural research.

In this paper, we reviewed four aspects of multi-sited and cross-cultural research that teams should consider as they plan and conduct such projects: team and project management; protocol development; data management and documentation; and equitable and collaborative practices. Throughout our discussion we have emphasized the importance of collaboration with local scientists and communities, open science practices, and early planning to the success of the project. In several places, we have highlighted considerations that could impact budgets and timelines. Meaningful, collaborative, and successful cross-cultural and multi-sited researches are necessarily ‘slower’ forms of research—this fact *must* be recognized, valued, and incentivized by funders and personnel review committees. We summarize the key takeaways of this article in figure 3.

Although our project was undertaken prior to Burger and colleagues' [12] call to develop cross-cultural data infrastructures in cognitive and behavioural research, our project procedures implemented several of their recommendations. Their call highlighted four main challenges which we have also highlighted: (1) protocol development workflows; (2) explicit/efficient data and quality assurance/quality control workflows; (3) researcher diversity; and (4) community inclusion. Burger *et al*. intended their paper to be the beginning of a broader conversation on how best to conduct cross-cultural research. Our paper can be seen as a response to this call, adding our experiences and insights to the discussion on what cross-cultural projects need to be successful.

Though our discussion has focused on cross-cultural research in the behavioural sciences, the lessons learned are transportable to other biological disciplines. All four aspects of cross-cultural research we have highlighted in this paper are important components of collaborative, multi-sited, international research that occurs in adjacent fields such as psychology, field biology, and ecology. Discussion that parallels the aspects we have highlighted is occurring in these fields, for example regarding data management and analysis workflows [27,36], open science practices [37,38], adaptation of data protocols for use across multiple countries, settings, ecologies and/or laboratories [8,37], as well as project and team management and collaborative practices [1]. Our recommendations will therefore be useful to researchers working on multi-site research in biological disciplines beyond the behavioural sciences.

To the existing discussion on best practices in cross-cultural research (e.g. [2,9–14,16]), this paper has added practical and technical considerations. For example, we explicitly discuss the importance of proper team and project management, introduce practical recommendations for exploring the validity of protocols through qualitative techniques during piloting, highlight the importance of good documentation at all steps of the project, and demonstrate how data management workflows can be strengthened through open science practices. Learning from our own cross-cultural project, we would recommend a generalized workflow that incorporates a range of perspectives on the research early on, dedicates meaningful time to pilot work, and involves planning for documentation, analysis, and repositing from the start. Open science principles and equity should be woven into all aspects of the project. In several places, we have also illustrated that funding agencies must recognize the unique needs of cross-cultural projects, including the need for relationship building, multiple and often long pilots, and investment in local researchers and communities that impact both budgets and timelines. As Savage and team [14] have written, we hope that in time, these recommendations ‘will seem to be so obvious as hardly worth stating’.

## Data Availability

This article has no additional data. The project materials and data used in specific analyses can be found on the Open Science Framework at: https://osf.io/mztep/. Electronic supplementary material is available online [39].
